# Treatment outcome of clonal cytopenias of undetermined significance: a single-institution retrospective study

**DOI:** 10.1038/s41408-021-00439-x

**Published:** 2021-03-01

**Authors:** Zhuoer Xie, Ahmad Nanaa, Antoine N. Saliba, Rong He, David Viswanatha, Phuong Nguyen, Dragan Jevremovic, Patricia Greipp, Mohamad E. Salama, Naseema Gangat, Hassan B. Alkhateeb, Ayalew Tefferi, Mark Litzow, Mrinal Patnaik, Mithun Shah, Aref Al-Kali

**Affiliations:** 1 Division of Hematology, Mayo Clinic, Rochester, MN USA; 2 Department of Laboratory Medicine and Pathology, Mayo Clinic, Rochester, MN USA; 3 Department of Hematopathology, Mayo Clinic, Rochester, MN USA

**Keywords:** Myelodysplastic syndrome, Cancer epigenetics

Dear Editor,

Clonal cytopenia(s) of undetermined significance (CCUS) is defined by the presence of the somatic mutation(s) of genes associated with myeloid neoplasms, with unexplained cytopenia(s) but without definitive morphologic evidence of myeloid neoplasms^1,2^. CCUS is a newly described entity with a high probability of progression to myeloid disorders upon follow-up^3,4^. However, there is a paucity of data on the efficacy of treatment^5^. We hereby describe a single-institution’s experience in managing CCUS patients who required treatment beyond blood transfusions.

This retrospective study was approved by scientific and ethical review boards at our institution. Patients who had CCUS and had received treatment beyond blood transfusions were identified from medical records. CCUS diagnosis was confirmed based on the absence of definitive morphologic evidence of myeloid neoplasms from bone marrow biopsy evaluation combined with evidence of pathogenic myeloid somatic mutation with a variant allele frequency (VAF) of at least 2% using our institution’s next-generation sequencing (NGS) panel (OncoHeme, Mayo Clinic)^6^. Treatment type and the indication were based on the treating physician’s choice. Clinical and laboratory results were collected. Hematologic improvement (HI) was graded based on the MDS International Working Group (IWG) 2006 criteria^7^. Symptomatic improvement (SI) was defined by the subjective improvement of CCUS-related inflammatory symptoms based on the patients’ report. The treatment response rate (RR) was calculated based on HI and SI. Progression was defined by either the occurrence of myeloid neoplasm or worsening cytopenia(s). Dependence on blood transfusion was defined as requiring an average of ≥2 units of packed red blood cells (RBCs) or platelets over 4 weeks or ≥4 units over 8 weeks. The date of treatment initiation was used for calculating overall survival (OS) and progression-free survival (PFS). Stata 14.1 (StataCorp) was used for data analysis.

Between July 2015 and July 2020, 24 patients met the inclusion criteria with a median age of 72 years (range: 24–87). The majority of patients were males (*N* = 20, 83%). Three (13%) patients had a history of the hematological condition (one monoclonal B cell lymphocytosis [MBL], one congenital neutropenia, and one acute myeloid leukemia [AML]) in remission). Three (13%) had a history of solid organ malignancy (one bladder cancer, one bronchogenic carcinoma, and one lung cancer). Two (8%) patients had prior radiation therapy, two (8%) had prior chemotherapy, and one (4%) had prior allogeneic stem cell transplantation. Six (25%) patients had inflammatory conditions (one Sweet’s syndrome, one periodic fever syndrome, one systemic inflammatory response syndrome, one poly-inflammatory syndrome with vasculitis, one myopericarditis, and one inflammatory purpura) (Table 1 and Supplemental Table 1). At the time of treatment, median hemoglobin was 8.9 g/dl (range 6.8–13.7), white blood cell 2.9 × 10^9^/l (range 1.4–12.3), and platelet 76 × 10^9^/l (range 8–407). RBC- and platelet transfusion-dependent rates were 54% and 29%, respectively. We identified 21 different mutations. Most common mutations occurred in *SRSF2* (*N* = 5, 21%), *TET2* (*N* = 5, 21%), *U2AF1* (*N* = 5, 21%), and *TP53* (*N* = 4, 17%) (Supplemental Fig. 1). This is consistent with previous reports that these genes are commonly seen in clonal hematopoiesis of indeterminate potential or CCUS^8,9^. Ten (42%) patients had 1 mutation, and 14 (58%) had ≥2 mutations. The median VAF was 25.7% (range 1.5–92%) (Supplemental Table 2).

**Table 1:** Patient characteristics with CCUS, the treatment regimens and outcomes.

Case no.	Age (years)	Sex	Clinical features	1st NGS-mutated gene (VAF%)	2nd NGS, if available (VAF%)	3rd NGS, if available (VAF%)	Treatment	Response	Follow-up duration (M)	Outcomes
1	60	M	Anemia and thrombocytopenia; Sweet syndrome	*TET2* (44%); *TET2* (41%)	—	—	HMA (DAC 5d); steroid	SI	6.7	Alive
2	71	M	Neutropenia; a history of chronic lymphocytic leukemia	*IDH1* (33%); *SRSF2* (39%)	—	—	HMA (DAC 3d)	HI	1.4	Alive
3	74	M	Neutropenia; acquired periodic fever syndrome	*IDH2* (37%); *ZRSR2* (74%)	—	—	HMA (DAC 3d)	SI	2.4	Alive
4	71	M	Anemia and thrombocytopenia; hypogammaglobinemia; systemic inflammatory response syndrome	*DNMT3A* (47%)	*DNMT3A* (44%)	—	HMA (DAC 3d)	HI	12.2	Alive
5	78	M	Pancytopenia	*U2AF1* (22%)	—	—	Steroid; IVIG; ESA	Stable	53.3	Alive
6	70	M	Pancytopenia; a history of bladder cancer	*TP53* (5%)	—	—	HMA (DAC 5d)	HI	9.6	Alive
7	73	M	Anemia	*SF3B1* (34%); *TET2* (8%)	—	*SF3B1* (32%); *IDH1* (48%)	Testosterone; ESA	MDS	87.8	Alive
8	78	M	Pancytopenia	*SF3B1* (missing)	—	—	Vitamin B_12_; iron	MDS	12.1	Alive
9	67	F	Neutropenia and thrombocytopenia	*RUNX1* (missing)	*BCOR* (27%); *KRAS* (23%); *U2AF1* (24%)	*KRAS* (38.9%); *U2AF1* (45.2%)	TPA	Initial HI, then AML	15.5	Alive
10	78	M	Anemia and thrombocytopenia; poly-inflammatory syndrome characterized by vasculitis; bronchogenic carcinoma	*KDM6A* (8%); *TET2* (22%); *U2AF1* (18%); *ZRSR2* (92%)	—	—	HMA (DAC 3d)	SI	2.3	Alive
11	64	M	Neutropenia and thrombocytopenia	*CUL3* (25.7%); *SRSF2* (43.5%); *TET2* (4.5%); *TET2* (1.5%)	*SRSF2* (43%); *TET2* (11%)	—	Rituximab	Stable	1.7	Alive
12	81	M	Neutropenia and thrombocytopenia	*IDH1* (12%); *SRSF2* (15%)	—	—	Steroid; GCSF	HI	6.6	Alive
13	88	M	Anemia	*ZRSR2* (34%)	—	—	Testosterone	Stable	48.3	Alive
14	74	M	Pancytopenia	*DDX41* (5%); *DNMT3A* (7%)	—	—	GCSF	Stable	3.5	Alive
15	24	M	Neutropenia and thrombocytopenia	*ASXL1* (32%)	—	—	GCSF; ESA	Initial HI, then progressed with worsening cytopenia (s)	18.5	Dead
16	76	M	Anemia and thrombocytopenia	*SRSF2* (32%); *TP53* (22%)	*TP53* (missing)	—	ESA	Stable	18.9	Alive
17	69	F	Pancytopenia; history of lung cancer	*JAK2* (2%); *TP53* (16%)	*JAK2* (10%); *TP53* (13%); *U2AF1* (6%)	—	Steroid; cyclosporine; ESA	Worsening cytopenia (s)	15.4	Dead
18	78	M	Pancytopenia	*U2AF1* (25%)	*ASXL1* (9%); *U2AF1* (18%)	—	ESA; testosterone	Stable	10.7	Alive
19	69	F	Anemia and neutropenia	*IDH1* (38%); *SRSF2* (43%); *TP53* (42%)	—	—	ESA	SI	22	Alive
20	75	M	Pancytopenia	*BCOR* (19%); *U2AF1* (9%)	—	—	Cyclosporine	Worsening cytopenia (s)	10.2	Dead
21	33	M	Pancytopenia; recurrent skin, soft tissue infection, diverticulitis; myopericarditis; history of congenital neutropenia	*SETBP1* (50%)^b^	—	—	Allogeneic stem cell transplantation	HI	32.6	Alive
22	58	F	Pancytopenia; irritable bowel syndrome; fibromyalgia; and inflammatory purpura without vasculitis	*ASXL1* (25%)	—	*ASXL1* (27%); *SETBP1* (12%); *SETBP1* (9%)	HMA (DAC 3d)	Initial HI, then MDS	26.3	Alive
23	77	M	Anemia and neutropenia;	*ASXL1* (missing); *U2AF1* (missing)	—	—	HMA (AZA^a^)	MDS	9	Alive
24	70	M	Pancytopenia; AML	*BCOR* (19%); *DNMT3A* (19%); *PHF6* (15%); *RUNX1* (9%); *SF3B1* (12%); *STAG2* (22%); *TET2* (9%)	*BCOR* (64%); *DNMT3A* (37%); *FLT3* (39%); *PHF6* (9%); *RUNX1* (32%); *SF3B1* (36%); *STAG2* (68%); *TET2* (32%)	—	HMA (AZA 3d)	AML	4.7	Alive

*NGS* next-generation sequencing, *VAF* variant allele frequency, *MDS* myelodysplasia neoplasm, *AML* acute myeloid leukemia, *SI* symptomatic improvement, *HI* hematologic improvement, *HMA* hypomethylating agents, *ESA* erythropoietin-stimulating agents, *GCSF* granulocyte-stimulating factor, *TPA* thrombopoietin receptor agonist, *IVIG* intravenous immunoglobulin, *DAC 5d* decitabine 5 days regimen, *DAC 3*d decitabine 3 days regimen, *AZA 3d* azacitadine 3 days regimen, *M* month.

^a^Missing details.

^b^Non-germline mutation.

Treatment included hypomethylating agents (HMAs) (*N* = 9, 38% [six treated with 3 days regimen], growth factors (*N* = 11, 46%) [erythropoietin-stimulating agents (ESA) (*N* = 7, 29%), granulocyte-stimulating factor) (*N* = 3, 13%) and thrombopoietin receptor agonist (*N* = 1, 4%)], steroids (*N* = 4, 17%), testosterone (*N* = 2, 8%), cyclosporine (*N* = 2, 8%), rituximab (*N* = 1, 4%), intravenous immunoglobulin (*N* = 1, 4%), and vitamin B_12_ and iron (*N* = 1, 4%). Seven (29%) patients received ≥2 different agents. The median time from diagnosis to treatment was 2.1 months (range 0–26.8) with a median follow-up duration of 14.3 months (range 2.3–59.9). The overall best RR was 50% (HI: *N* = 8 (33%) and SI: *N* = 4 (17%)). Three (13%) patients subsequently progressed after the initial response. Most responders (58%) were treated with HMA (RR = 78%, *p* = 0.04 [HI: *N* = 3, RR = 57% and SI: *N* = 3, RR = 43%]) with a median response duration of 6.8 months (range: 1.4–12.4) (Supplemental Figure 2)*.* HMA RR was not associated with mutations in DNA methylation genes (*p* > 0.05). Among the patients with inflammatory conditions, five were treated with HMA, and the SI-RR was 60%. The RR was 100% for the three patients with *IDH1* mutation (treated with HMA, steroids, and ESA, respectively). The RR was 67% for patients with *ZRSR2* mutation (two HMA, one testosterone). The RR was 0% for three patients with *SF3B1* mutation (one testosterone and ESA, one vitamin B_12_ and iron, and one HMA) (Table 1). None of the responders had *ASXL1* mutation. Figure 1 demonstrates the RR for each treatment. Patients with a single mutation (compared to ≥2 mutations) were significantly more likely to achieve HI (RR 60 vs. 14%, *p* = 0.03). At the data cutoff, RBC and platelet transfusion-dependent rate were 38% (30% reduction) and 25% (14% reduction), respectively.

**Fig. 1:**
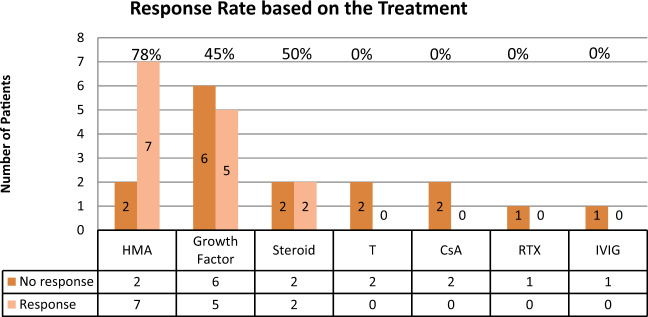
Disease response rate (RR). Number and percentage of responders based on the treatment types. HMA hypomethylating agents, T testosterone, CsA cyclosporin, IVIG intravenous immunoglobulin, RTX rituximab.

Six patients (25%) progressed to myeloid malignancy (four MDS and two AML). Five (21%) had worsening cytopenias. Among the four MDS patients, they had *SF3B1*, *TET2*, *ASXL1*, and *U2AF1* mutations at the diagnosis of CCUS. Within the two AML patients, both had *RUNX1* mutation (Table 1 and Supplemental Table 3). A recent report from Malcovati et al.^10^ suggests that *SF3B1* mutation in CCUS represents a distinct MDS entity. All three *SF3B1* mutant CCUS patients progressed to myeloid neoplasms in this cohort. Two patients with a prior diagnosis of solid organ malignancies (treated with cytotoxic chemotherapy) had CCUS with *TP53* mutation. One patient treated with HMA achieved HI, while the other, treated with steroids, cyclosporine, and ESA, did not respond. The median PFS was 17.1 months (95% CI: 7.1–not reached). The median OS was not reached with an estimated 2-year OS of 73% (Supplemental Figure 3A, B). Three (13%) patients had worsening cytopenias leading to death (one septic shock, two transitioned to hospice care). These three patients’ molecular signature at CCUS diagnosis showed *ASXL1*, *TP53*, and *U2AF1* mutations, respectively (Table 1 and Supplemental Table 4). The 30- and 60-day mortality was 0%.

Subsequent NGS was available for nine (38%) patients. Six (66.7%) had a clonal evolution (acquiring new mutations). Among these, two progressed to AML (one had new *BCOR*, *KRAS*, and *U2AF1* mutations, one had new *FLT3* mutation); two progressed to MDS (one had new *IDH1* mutation, one had new *SETBP1* mutation); one had worsening cytopenia (new *U2AF1* mutation); one had stable disease (new *ASXL1* mutation); two had stable disease with no clonal evolution; one responded to HMA treatment with the *DNMT3A* VAF decreasing from 47 to 44% (Table 1 and Supplemental Table 3). In our cohort, five (83%) clonal evolution co-occurred with the progression of myeloid neoplasm.

In summary, this is the first report of the outcome of treatment in CCUS patients requiring therapies beyond blood transfusion. Our results suggest that patients with CCUS responded to available treatments for myeloid disorders and immune cytopenias. HMA was safe and associated with the highest RR and CCUS-associated inflammatory symptoms in this cohort. Our data also suggest that the mutation profile may impact treatment response. Several limitations exist in our study. First, the retrospective nature of our cohort and the relatively small sample size limit the generalizability of our observations. Second, there were heterogeneities in both patients and therapies. Three patients with pre/co-existing hematological conditions (one MBL, one congenital neutropenia who received stem cell transplantation for pancytopenia, and one AML in remission) were included as they did not fulfill myeloid malignancy criteria. Several patients were diagnosed with inflammatory disorders or malignancies and received prior cytotoxic chemotherapy making them a higher risk CCUS. Although CCUS is associated with high inflammatory status^11^, it is unclear if the immune-mediated marrow process is the driver of cytopenia or the clonal process itself. The therapies also varied based on the treating physician’s decision and were adopted from myeloid disorder or immune-related cytopenia as no standard of care exists to date. Third, the best treatment response was defined as both hematological and symptomatic responses due to the unique features of this cohort, with inflammatory status present in a quarter of our patients. Last, the MDS IWG criteria were used to assess hematological response for this cohort due to a lack of other criteria. An international multicenter study is ongoing to validate our current findings and determine the clinical outcome for treatment in CCUS. Future studies are warranted to develop response and progression criteria to facilitate and standardize reporting treatment outcomes.

## Supplementary information

Supplementary tables
