# Supplementary material for: Treatment outcome of clonal cytopenias of undetermined significance: a single-institution retrospective study
Source: Blood Cancer J. 2021 Mar 1;11(3):43. doi: 10.1038/s41408-021-00439-x (PMC7921651; doi:10.1038/s41408-021-00439-x)
Supplement: Supplementary file 1 — Supplementary tables [file 41408_2021_439_MOESM1_ESM.docx]

**Supplemental Table 1. Sociodemographic and Clinical Data**

| **Variable** |  |
| --- | --- |
| Age, Median (Range) | - 72 (24-87) |
| Sex, N (%) | - Female: 4 (17%) - Male: 20 (83%) |
| Race, N (%) | - White: 15 (63%) - African American: 1 (4%) - Native American: 1 (4%) - Unknown: 7 (33%) |
| Smoking history, N (%) | - Current smoker: 3 (13%) - Former smoker: 11 (46%) - Never smoker 10 (42%) |
| Body Mass Index, mean (SD) | - 27.9 (4.9) |
| Number of comorbidities, N (%) | - 0 comorbidity: 4 (17%) - 1 comorbidity: 4 (17%) - 2 comorbidities: 5 (21%) - 3 comorbidities: 4 (17%) - ≥4 comorbidities: 7 (29%) |
| ECOG Performance status, N (%) | - ECOG PS 0: 6 (25%) - ECOG PS 1: 9 (38%) - ECOG PS 2: 4 (17%) - Missing: 5 (21%) |
| Hemoglobin, median (ranges) | - 8.9 (6.8-13.7) gram/dicelitre |
| White blood cell, median (ranges) | - 2.9 (1.4-12.3) *10^9^/litre |
| Platelet, median (ranges) | - 76 (8-407) *10^9^/litre |
| Variant Allele Frequency, median (ranges) | - 25.7% (1.5%-92%) |
| Treatment, N (%) | - HMA: 9 (38%) - Growth factor: 11 (46%) - Steroid: 4 (17%) - Testosterone: 2 (8%) - Cyclosporine: 2 (8%) - Rituximab: 1 (4%) - IVIG: 1 (4%) - Vitamin B12 and iron: 1 (4%) |

Abbreviations: HMA, hypomethylating agents; GCSF, granulocyte stimulating factor; IVIG, intravenous immunoglobulin

**Supplemental Table 2: Pathogenic Mutation Frequency and the Variant Allele Frequency Value**

| **Gene** | **N** | **Mean, %(SD)** | **Minimal, %** | **Maximum, %** |
| --- | --- | --- | --- | --- |
| *ASXL1* | 3*** | 28.5 (4.9) | 25 | 32 |
| *BCOR* | 2 | 19 (0) | 19 | 19 |
| *CUL3* | 1 | 25.7 | - | - |
| *DDX41* | 1 | 5 | - | - |
| *DNMT3A* | 3 | 24.5 (20.5) | 7 | 47 |
| *IDH1* | 3 | 27.7 (13.8) | 12 | 38 |
| *IDH2* | 1 | 37 | - | - |
| *JAK2* | 1 | 2 | - | - |
| *KDM6A* | 1 | 8 | - | - |
| *PHF6* | 1 | 15 | - | - |
| *RUNX1* | 2*** | 9 | - | - |
| *SETBP1* | 1 | 50 | - | - |
| *SRSF2* | 5 | 34.5 (11.8) | 15 | 43.5 |
| *SF3B1* | 3*** | 23 (15.6) | 12 | 34 |
| *STAG2* | 1 | 22 | - | - |
| *TET2* | 5 | 17.5 (16.2) | 4.5 | 44 |
| *TP53* | 4 | 21.3 (15.5) | 5 | 42 |
| *U2AF1* | 4 | 18.5 (7) | 9 | 25 |
| *ZRSR2* | 3 | 66.7 (30) | 34 | 92 |

*1 missing value

**Supplemental Table 3: Initial and Subsequent Genetic Profile based on the Outcomes**

| **Disease** | **Case Number** | **Initial mutation🡪 subsequent mutation if available** | **Treatment** |
| --- | --- | --- | --- |
| AML | 9* | *RUNX1🡪 BCOR; KRAS; U2AF1* | TPA |
|  | 24 | *BCOR; DNMT3A; PHF6; RUNX1; SF3B1; STAG2; TET2🡪*  *BCOR; DNMT3A; FLT3; PHF6; RUNX1; SF3B1; STAG2; TET2* | HMA |
| MDS | 7 | *SF3B1; TET2🡪SF3B1; IDH1* | Testosterone and ESA |
|  | 8 | *SF3B1* | Vitamin B12 and iron |
|  | 22* | *ASXL1🡪 ASXL1; SETBP1* | HMA |
|  | 23 | *ASXL1, U2AF1* | HMA |
| Worsening cytopenia(s) | 15* | *ASXL1, subsequently progress* | GCSF; ESA |
|  | 17 | *JAK2, TP53*🡪 *JAK2; TP53; U2AF1* | Steroid; Cyclosporine; ESA |
|  | 20 | *BCOR; U2AF1* | Cyclosporine |
| Stable | 5 | *U2AF1* | Steroid; IVIG; ESA |
|  | 11 | *CUL3; SRSF2; TET2*2🡪* *SRSF2; TET2* | Rituximab |
|  | 13 | *ZRSR2* | Testosterone |
|  | 14 | *DDX41; DNMT3A* | GCSF |
|  | 16 | *SRSF2; TP53🡪* *TP53* | ESA |
|  | 18 | *U2AF1*🡪 *ASXL1; U2AF1* | ESA; Testosterone |
| Symptoms improvement | 1 | *TET2*2* | HMA; Steroid |
|  | 3 | *IDH2; ZRSR2* | HMA |
|  | 10 | *KDM6A; TET2; U2AF1; ZRSR2* | HMA |
|  | 19 | *IDH1; SRSF2; TP53* | ESA |
| Hematologic improvement | 2 | *IDH1; SRSF2* | HMA |
|  | 4 | *DNMT3A🡪 DNMT3A* | HMA |
|  | 6 | *TP53* | HMA |
|  | 12 | *IDH1; SRSF2* | Steroid; GCSF |
|  | 21 | *SETBP1* | Allogeneic stem cell transplantation |

**Abbreviations:** MDS, myelodysplasia neoplasm; AML, acute myeloid leukemia; HMA, hypomethylating agents; ESA, erythropoietin stimulating agents; GCSF, granulocyte stimulating factor; TPA, thrombopoietin receptor agonist; IVIG, intravenous immunoglobulin. *cases initially responded to the treatment but subsequently progressed.

**Supplemental Table 4: Pathogenic Mutation, Co-occurring Status and their Outcomes**

|  | **Pathway** | **N. of mutated patients** | | | **MDS/AML** | **Worsening**  **cytopenia(s)** | **Hematologic improvement** | **Symptoms improvement** | **Stable** |
| --- | --- | --- | --- | --- | --- | --- | --- | --- | --- |
|  |  | Total number | Isolated N (%) | Co-Mutated N, (%) |  |  |  |  |  |
| *SRSF2* | RNA splicing | 5 | - | 5, (100%) | - | - | 2, (40%) | 1, (20%) | 2, (40%) |
| *TET2* | DNA methylation | 5 | 1, (20%) | 4, (80%) | 2, (40%) | - | - | 2, (40%) | 1, (20%) |
| *TP53* | Tumor suppressor | 4 | 1, (25%) | 3, (75%) | - | 1, (25%) | 1, (25%) | 1, (25%) | 1, (25%) |
| *U2AF1* | RNA splicing | 5 | 2, (40%) | 3, (60%) | 1, (20%) | 1, (20%) |  | 1, (20%) | 2, (40%) |
| *IDH1* | DNA methylation | 3 | - | 3, (100%) | - | - | 2, (66.7%) | 1, (33.3%) | - |
| *ZRSR2* | RNA splicing | 3 | - | 3, (100%) | - | - |  | 2, (66.7%) | 1, (33.3%) |
| *DNMT3A* | DNA methylation | 3 | 1, (33.3%) | 2, (66.7%) | 1, (33.3%) | - | 1, (33.3%) | - | 1, (33.3%) |
| *SF3B1* | RNA splicing | 3 | 1, (33.3%) | 2, (66.7%) | 100% | - | - | - | - |
| *RUNX1* | Transcription regulation | 2 | 1, (50%) | 1, (50%) | 100% | - | - | - | - |
| *ASXL1* | Chromatin & Histones modifier | 3 | 2, (66.7%) | 1, (33.3) | 2, (66.7%) | 1, (33.3%) | - | - | - |
| *BCOR* | Chromatin & Histones modifier | 2 | - | 2, (100%) | 1, (50%) | 1, (50%) | - | - | - |
| *IDH2* | DNA methylation | 1 | - | 1, (100%) | - | - | - | 1, (100%) | - |
| *KDM6A* | Chromatin & Histones modifier | 1 | - | 1, (100%) | - | - | - | 1, (100%) | - |
| *CUL3* | Signaling | 1 | - | 1, (100%) | - | - | - | - | 1, (100%) |
| *DDX41* | Tumor suppressor | 1 | - | 1, (100%) | - | - | - | - | 1, (100%) |
| *JAK2* | Signaling | 1 | - | 1, (100%) | - | 1, (100%) | - | - | - |
| *SETBP1* | Epigenetic regulator | 1 | 1, (100%) | - | - | - | 1, (100%) | - | - |
| *PHF6* | Tumor suppressor | 1 | - | 1, (100%) | 1, (100%) | - | - | - | - |
| *STAG1* | Cohesin complex | 1 | - | 1, (100%) | 1, (100%) | - | - | - | - |

**Abbreviations:** MDS, myelodysplasia neoplasm; AML, acute myeloid leukemia.

**Supplemental Figure 1**

**Pathogenic Mutation Frequency**

**Supplemental Figure 2**

**
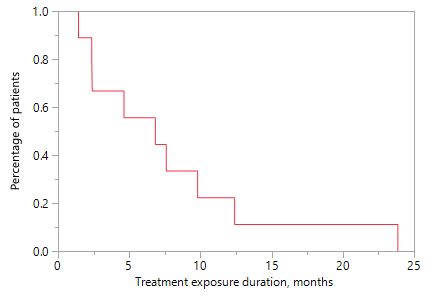
**

**Duration of hypomethylating agents exposure.**

**Supplemental Figure 3**

**A B**

**
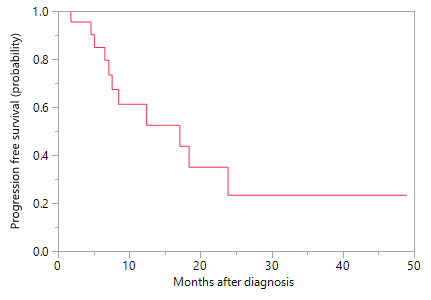

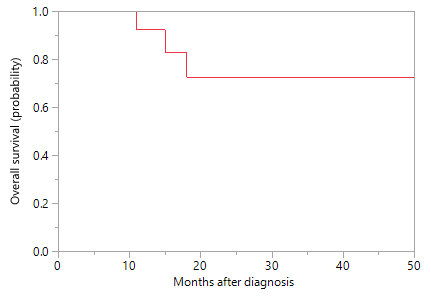
**

**Progression Free Survival (PFS) and Overall Survival (OS).** Kaplan-Meier estimates for the probability of (A) PFS and (B) OS.
